# Aβ12‐28P administration alleviates anxiety in an Alzheimer's disease mouse model

**DOI:** 10.1002/alz70859_105359

**Published:** 2025-12-26

**Authors:** Xiaoyi Yang, Zhuowei Gong, Fangzheng Tian, Karen Ha, Dmitri Lyalikov, Zedong Sun, Chris Kim, Tyra Nguyen, Yvonne Xu, Jasper Kamb, Amy Chen, Michael Xiao, Arjun V. Masurkar, Thomas Wisniewski, Shuo Chen

**Affiliations:** ^1^ NYU Grossman School of Medicine, New York, NY USA; ^2^ New York University, New York, NY USA; ^3^ New York University Grossman School of Medicine, New York, NY USA

## Abstract

**Background:**

Anxiety is a common symptom of Alzheimer's disease (AD). It has been recently established that anxiety is associated with AD pathology characterized by amyloid‐β (Aβ) plaques and neurofibrillary tangles. Our laboratory has demonstrated that acute administration of Aβ12‐28P, a synthetic peptide with the potential to competitively inhibit toxic Aβ oligomers, resulted in improved cognitive function in old transgenic AD mice. However, whether inhibiting Aβ toxicity by Aβ12‐28P also alleviates anxiety in AD mice remains unknown.

**Method:**

Middle‐aged (12‐15 months old) and old (18‐24 months old) APP/PS1 dE9 mice were intraperitoneally injected with Aβ12‐28P, while control mice received an equivalent volume of saline. Behavioral tests, including open field test and elevated plus maze, were conducted 2 hours post‐injection to assess the acute effect of Aβ12‐28P or saline on the anxiety of animals. We further performed in vivo fiber photometry to investigate anxiety‐associated neural dynamics across multiple brain regions, such as ventral CA1 (vCA1), medial prefrontal cortex (mPFC), basal amygdala (BA), and lateral hypothalamic area (LHA). Adeno‐associated virus expressing GCaMP8f was injected into target brain regions, above which an optic fiber was implanted. Four weeks later real‐time calcium transients were recorded during anxiety tests.

**Result:**

In middle‐aged AD mice, Aβ12‐28P administration exerted acute anxiolytic effects in the open field test (*n* = 8 drugged versus 8 non‐drugged, *p* < 0.05). Furthermore, anxiety was also significantly alleviated by Aβ12‐28P in old AD mice in both the open field (*n* = 17 drugged versus 14 non‐drugged, *p* < 0.0001) and elevated plus maze (*n* = 11 drugged versus 12 non‐drugged, *p* < 0.05) tests. We further compared the neural activity patterns in brain regions, such as vCA1, mPFC, BA, and LHA, with or without Aβ12‐28P treatment by fiber photometry. Our data suggested significant modification of anxiety‐associated neural dynamics in these brain regions by Aβ12‐28P administration.

**Conclusion:**

These results suggest that Aβ12‐28P has an acute anxiolytic effect in both middle‐aged and old AD mice. Our finding opens the possibilities to apply the strategy of targeting Aβ pathology for alleviating neuropsychiatric AD symptoms, such as anxiety, and provide insights into a brain region‐specific mechanism behind this therapeutic strategy.

